# Natural Anti-Inflammatory Agents in the Chemoprevention of Gliomas: Targeting Neuroinflammation and the Tumor Microenvironment

**DOI:** 10.3390/cancers17243922

**Published:** 2025-12-08

**Authors:** Georgios S. Markopoulos, Chrissa Sioka, George A. Alexiou, Dimitrios Peschos, George Vartholomatos, Athanassios P. Kyritsis

**Affiliations:** 1 Laboratory of Physiology, Faculty of Medicine, School of Health Sciences, University of Ioannina, 45110 Ioannina, Greece; gmarkop@uoi.gr (G.S.M.); dpeschos@uoi.gr (D.P.); 2 Neurosurgical Institute, Faculty of Medicine, School of Health Sciences, University of Ioannina, 45110 Ioannina, Greece; csioka@uoi.gr (C.S.); galexiou@uoi.gr (G.A.A.); 3 Laboratory of Haematology, Unit of Molecular Biology and Translational Flow Cytometry, University Hospital of Ioannina, 45500 Ioannina, Greece; gvarthol@gmail.com

**Keywords:** glioma, neuroinflammation, tumor microenvironment (TME), phytochemicals, cytokine modulation, oxidative stress, blood–brain barrier (BBB), antitumor natural compounds, neuroprotective agents, gut–brain–tumor axis

## Abstract

Chronic inflammation is a well-established hallmark of cancer, playing a critical role in the initiation and progression of gliomas. Recent evidence underscores the importance of anti-inflammatory natural products as chemotherapeutic and potentially as chemopreventive agents, offering a safe and multifaceted approach to mitigate tumor-promoting inflammation in the brain. This review explores the interplay between major inflammation-related pathways—such as NF-κB, COX-2, and the NLRP3 inflammasome—and key bioactive compounds derived from natural sources such as polyphenols, isothiocyanates, terpenes/lignans, and omega-3-derived mediators. We provide evidence on the effect of these compounds on the above inflammatory triangle. We discuss emerging in vitro, in vivo preclinical and translational evidence in the context of glioma biology and highlight how these compounds may pass the blood–brain barrier (BBB) and modulate the tumor microenvironment (TME), including immune cell infiltration and cytokine profiles that may act in a pro- or anti-inflammatory manner, highlighting their capacity to inhibit GBM-associated inflammation. Each substance may differentially influence the components of the inflammatory triangle. Overall, we position these agents as low-toxicity, formulation-aware adjuncts to standard care. The ultimate goal is offering novel insights on low-toxicity, inflammation-targeting interventions against malignant brain tumors.

## Simple Summary

Gliomas, including glioblastoma (GBM), are aggressive brain tumors in which chronic inflammation helps cancer cells grow, invade, and evade immunity. This review focus on three connected inflammatory circuits, NF-κB, COX-2–prostaglandin E2 (EP2/EP4), and the NLRP3–IL-1 pathway. This inflammatory triangle may shape tumor behavior and the surrounding immune microenvironment. We summarize evidence that diet-derived natural anti-inflammatory agents (polyphenols such as curcumin, resveratrol, EGCG, quercetin; isothiocyanates such as sulforaphane; terpenes/lignans such as boswellic acids and honokiol; ω-3-derived mediators; and berberine) may affect these signals, reduce invasion, and improve sensitivity to radiotherapy or temozolomide in experimental models. We summarize that these compounds may act as low-toxicity adjuncts to standard care, ideally using brain-directed formulations and biomarker monitoring based on their well-established anti-inflammatory and antioxidant roles.

## 1. Introduction

### 1.1. Introduction on Cancer, Brain Cancer, and Inflammatory Response

Globally, cancer is a leading cause of human mortality. According to the Global Burden of Disease Study, new cancer cases doubled since 1990 to 18.5 million and deaths rose to 10.4 million in 2023, while by 2050 there is an estimation for 30.5 million new cases and over 18 million deaths each year [1]. In 2023, an estimated 41.7% of cancer deaths were attributable to modifiable risk factors, underscoring the potential for prevention to curb the future burden [1]. Primary malignant brain tumors remain among the most lethal types of cancer, with glioma and high-grade glioblastoma (GBM) accounting for the majority of deaths, despite recent advances in surgery, radiotherapy, and systemic therapies [2,3,4]. Based on population data, five-year survival for GBM remains low, near 7%, underscoring an urgent need for preventive and adjunct strategies that occur before or during early disease [4]. Additionally, among all primary malignant brain and other CNS tumors, the overall five-year relative survival remains only at ~36%, highlighting the present limits of the current standards of care [5]. For diffuse gliomas, guidelines continue to recommend a combination of resection of maximal safe margins, tumor irradiation, and alkylating chemotherapy adapted to molecular subtype, based on the 2021 WHO classification [6,7]. There are several updated practice frameworks, such as EANO, RANO/EANO PET guidance, and NCCN that aim to refine diagnostics, response assessment, and adjuvant options. However, for most patients, diffuse gliomas are a devastating disease [7,8,9].

Targeting inflammatory responses associated with gliomagenesis and progression of the disease has been proposed as an additional treatment for brain tumors [10]. Inflammation has been extensively studied in several types of cancer and has emerged in the modern adaptation of the conceptual framework of “hallmarks of cancer” as an enabling characteristic and a therapeutic target [10]. In gliomas, the transcriptional programs that are affected by inflammation include three regulators. The transcription factor NF-κB [11], cyclooxygenase-2 (COX-2)–PGE_2_ signaling via the EP2/EP4 receptors [12], and the NLRP3 inflammasome [13]. NF-κB, COX-2, and PGE_2_ have been shown to cooperate to promote proliferation, invasion, angiogenesis, and escape of immune surveillance. These pathways are active in tumor cells and across the tumor microenvironment (TME). In particular, tumor-associated microglia and macrophages (collectively TAMs) may create positive feedback loops. These loops sustain an inflammation-dependent immunosuppressive niche [14]. NF-κB signaling is frequently upregulated in GBM and connects inflammatory stimuli in cells with gene expression patterns that enhance survival, stemness, and treatment resistance [15]. Interestingly, NF-kB-related networks have been shown to be attractive targets for natural product-related therapies [16]. In addition, both canonical and non-canonical NF-κB signaling intersect with signaling pathways such as those of TGF-β, PI3K/Akt, and MAPK that altogether amplify a pro-tumorigenic, proliferative signaling within the TME [11]. Elevated COX-2 and PGE_2_ can further affect TAMs to act in an immunosuppressive manner. Since such action correlates with aggressive disease, both COX-2 and PGE_2_ may be considered as rational targets in GBM [12,17]. Furthermore, NLRP3 activation in gliomas has context-dependent effects. Recent evidence links NLRP3 signaling to granulocytic recruitment in the TME and therapy antagonism, further supporting that inflammasome and tumor-promoting inflammation modulation may have strong antiglioma action [10].

### 1.2. Problem Definition and Scope

This narrative review focuses on adult diffuse gliomas and glioblastoma (GBM) and evaluates how NF-κB, COX-2–PGE_2_ (EP2/EP4), and NLRP3–IL-1 signaling intersect with natural anti-inflammatory compounds to shape the TME. GBM incidence increases with age and is modestly higher in males, contextualizing the population at risk. Key prognostic/predictive markers include *IDH* mutation, 1p/19q codeletion, and MGMT promoter methylation, which guide contemporary management and trial stratification. Our scope is gliomas not non-CNS cancers, and we emphasize translational relevance (delivery/BBB/BTB, biomarker readouts) rather than extrapolation [7,8,9,18].

The use of natural anti-inflammatory compounds, such as diet-derived or phytochemical agents may combine potential anticancer activity with a higher tolerability profile [19,20]. Many of these molecules seem to converge on the same inflammatory pathways that are aberrant in glioma, which include the activation of NF-κB, COX-2, and NLRP3, providing a mechanistic basis for potential chemopreventive and adjunct therapeutic roles [12,19,21]. Pleiotropic actions may simultaneously influence redox balance, epigenetic regulation, and metabolic signaling, properties that are advantageous in genetically heterogeneous tumors [11].

Importantly, the glioma TME is densely populated by TAMs and other myeloid cells, often comprising up to half of the live cells within GBM, and these cells are highly responsive to inflammatory stimuli [3]. Recent literature reviews detail the heterogeneity of microglia versus infiltrating macrophages and outline therapeutic concepts aimed at reprogramming the roles of these cell populations rather than depleting them [14,22,23]. Many natural agents modulate macrophage polarization, cytokine secretion, and prostaglandin pathways; they may influence the TME toward an antitumor phenotype when paired with standard therapy [12,24].

The gut–brain–tumor axis adds another layer of complexity and a connection between inflammation and tumor promotion. Emerging data suggest that gut microbial composition and metabolites regulate systemic and neuroinflammatory responses, with preliminary evidence linking dysbiosis to brain tumor biology and therapy responses [25,26]. Observational studies and meta-analyses indicate that healthier dietary patterns, rich in vegetables, fruits, fish, and whole grains, associate with lower glioma risk, though study heterogeneity remains [27,28,29]. A clear causality for specific protective dietary factors in glioma has not been established yet, underscoring the need for novel experimental designs [30]. Nevertheless, preclinical and early translational works suggest that dietary modulation (including low-carbohydrate paradigms) and microbiome-associated strategies may attenuate neuroinflammation and interact with antitumor immunity in glioma models, while gut dysbiosis promotes glioma [31,32].

From a safety and feasibility standpoint, several natural compounds commonly discussed in neuro-oncology contexts, such as curcumin and resveratrol, have been examined across non-glioma clinical settings and generally display acceptable tolerability at commonly studied oral doses, with bioavailability as a key limitation [33,34]. Umbrella and recent systematic reviews emphasize that while safety is encouraging, efficacy signals are inconsistent and often limited by small, heterogeneous trials and formulation issues [33,35]. These realities argue for adjunct rather than replacement roles and motivate formulation innovations, such as nano-delivery and phytosomes, to improve brain exposure and pharmacokinetics [6].

Within this review, we discuss the interplay of natural anti-inflammatory compounds with three core inflammatory compounds in glioma, namely, NF-κB, COX-2, and NLRP3, and how these interactions might reprogram the TME to reduce gliomagenesis, immunosuppression, and invasion. We frame these compounds as low-toxicity, inflammation-targeted complements to standard care rather than as stand-alone cures, with a goal of improving quality of life and delaying progression and, in the future, of identifying possible subgroups for which preventive or adjunct approaches are most impactful.

### 1.3. Methods—Scope of the Literature Survey

The current work is a narrative review. We searched PubMed and Scopus for English-language reports (2015–2025, plus earlier foundational studies) using combinations of glioma OR glioblastoma AND (NF-κB OR COX-2 OR PGE_2_ OR EP2 OR EP4 OR NLRP3 OR IL-1β) AND (polyphenols OR sulforaphane OR honokiol OR ginsenosides OR ω-3 OR specialized pro-resolving mediators OR berberine). We prioritized mechanistic and orthotopic in vivo evidence and recent syntheses for broader context. References were selected to illustrate mechanisms, translational constraints (BBB/BTB, formulation), and adjunctive potential not to exhaustively catalogue all publications.

## 2. Inflammation in Glioma: Core Components

It is known that gliomas, especially glioblastoma multiforme, are sustained by inflammatory circuitry that may integrate tumor-promoting signals with the TME interplay. Among the core components of inflammation are NF-κB-associated signaling, the COX-2/PGE_2_ cascade, and NLRP3 inflammasome activation, which together may influence proliferation, invasion, angiogenesis, and immune evasion [3,36,37]. Within the modern “hallmarks of cancer” framework, tumor-promoting inflammation is an enabling characteristic, making these pathways important for possible therapeutic interventions [10].

### 2.1. NF-κB Signaling: A Central Inflammatory Integrator in Glioma

The NF-κB pathways are commonly stress- and cytokine-responsive signaling cascades in which IκB kinases (IKKα/IKKβ with NEMO) phosphorylate IκB inhibitors, targeting them for degradation and freeing NF-κB transcription factor dimers (commonly p65/RelA–p50) to translocate to the nucleus. There, NF-κB drives transcription of a wide array of target genes. Based on the initial signal, the specific dimer activated, and the cell type, NF-κB can induce inflammation, immunity, cell survival, and metabolism. A tight negative feedback (such as IκBα resynthesis) and alternative (non-canonical) signaling via NIK–IKKα that processes the p100 subunit to p52 can also fine-tune context-dependent responses [11].

Canonical NF-κB signaling, typically based on IKKβ-dependent phosphorylation and degradation of IκBα, enabling p65/p50 nuclear translocation, and non-canonical signaling, NIK-IKKα-dependent processing of p100 to p52/RelB, are both engaged in glioma, integrating cytokines (e.g., TNFα, IL-1β), DAMPs, and receptor tyrosine kinase activity [38,39]. Aberrant activation has been shown to support tumor-cell survival, metabolic adaptation, angiogenesis, invasion, and therapy resistance, while it may also contribute to immunosuppression within the TME [14,38]. Recent research highlights that NF-κB may act towards GBM pathogenesis, with upstream drivers including the EGFR/PI3K/Akt pathway, TGF-β signaling, and extracellular matrix remodeling. Downstream effectors may include cytokines, chemokines, and EMT-based transcriptional responses [40]. Experimental studies have established a link between NF-κB activity and chemotherapy resistance. An interesting example includes the RIP2/NF-κB/PD-L1-driven induction of autophagy following temozolomide treatment, showing a role in treatment failure, enforced by in vitro and xenograft GBM models [41]. At the TME level, NF-κB signaling in tumor-associated microglia/macrophages (TAMs) shapes pro-inflammatory cytokine expression and antigen presentation, biasing toward an immune-evasive local environment [14]. Through crosstalk with STAT3, MAPKs, and PI3K/Akt, NF-κB amplifies glioma stem-like cells, their invasion, and resistance to radiation and temozolomide [38]. Collectively, these data support that NF-κB modulators may have tumor suppressor roles in GBM. Towards this direction, a recent work by our team has introduced an NF-κB-related network that is active during the antiglioma action of natural compounds deglucohellebrin, n-p-cumaroyl-serotonine, and moschamine [16]. This network explains the antiglioma role of each of these substances [42,43,44].

### 2.2. COX-2/PGE_2_: Prostaglandin Signaling and Immune Evasion

Cyclooxygenase-2 (COX-2/PTGS2) catalyzes arachidonic acid conversion to prostaglandins, with PGE_2_ acting via EP receptors (EP1–EP4). In cancer, EP2/EP4 are key mediators of immunosuppression and tumor progression [45]. In glioma, COX-2 is frequently upregulated in tumor cells and myeloid populations, and PGE_2_ signaling contributes to growth, angiogenesis, and immune evasion [46]. Recent work shows that the COX-2/PGE_2_ axis reshapes the GBM TME and alters glioma-associated microglia/macrophage (GAM/TAM) dynamics, sustaining pro-tumorigenic cytokine loops that promoting vascular changes in the TME [46].

Mechanistically, PGE_2_ signals through EP2 and EP4 receptors, and dual or selective blockade of either one reduces growth, angiogenesis, and immune evasion in preclinical models of malignant glioma [47]. Beyond tumor-cell intrinsic effects, PGE_2_ drives myeloid-derived suppressor cell (MDSC) differentiation and suppressive programming, largely via EP2/EP4, which limits antitumor T- and NK-cell activity [45,48]. In glioma, PGE_2_-EP4 signaling has been specifically tied to inhibition of NK-cell function and IFN-γ production, further consolidating a role in immune evasion [49].

The result of elevated COX-2/PGE_2_ signaling is a TME that leans toward immunosuppression, enhanced invasion via matrix remodeling and EMT-associated signaling, and a pro-angiogenic phenotype in GBM models [46,47].

### 2.3. NLRP3 Inflammasome: IL-1 Family Signaling and Myeloid Cell Populations

The NLRP3 inflammasome is activated through a two-step process involving priming and activation. During the priming phase, signals such as pathogen-associated or damage-associated molecular patterns activate NF-κB, which induces the transcription of NLRP3, pro–IL-1β, and pro–IL-18, thereby increasing the availability of key inflammasome components. The subsequent activation step is triggered by a wide range of stimuli, including potassium efflux, mitochondrial dysfunction, or lysosomal damage, that promote NLRP3 oligomerization and its assembly with the adaptor protein ASC and the effector protease caspase-1. This assembled complex enables the autocatalytic activation of caspase-1, leading to the cleavage and maturation of the pro-inflammatory cytokines IL-1β and IL-18, and the induction of pyroptotic cell death [50]. In the CNS, NLRP3 links signals like DAMPs, ROS, ionic flux to neuroinflammation. in tumors, its effects are context-dependent and can be pro- or antitumorigenic [50].

Accumulating recent evidence suggests that NLRP3 contributes to glioma progression by enabling an immunosuppressive microenvironment and facilitating invasion [51]. Notably, NLRP3 activation expands granulocytic populations, inhibits antitumor immunity, and antagonizes STING-pathway therapy in GBM models [52]. Clinically relevant cytokine loops can also emerge. IL-1β-positive TAMs can amplify PGE_2_-EP4 signaling, which in turn accelerates GBM progression and provides a link between inflammasome activity and prostaglandin-driven immune evasion [53]. Through IL-1β/IL-18 and downstream chemokines, NLRP3 signaling promotes myeloid recruitment, T-cell dysfunction, and tissue remodeling that support invasion [14,52]. While in some tumors inflammasome activity may act as an antitumor mechanism, current GBM data show a possible pro-tumorigenic, immunosuppressive action [13,50].

### 2.4. Crosstalk and Positive-Feedback Loops Across NF-κB, COX-2/PGE_2_, and NLRP3

**Figure 1:**
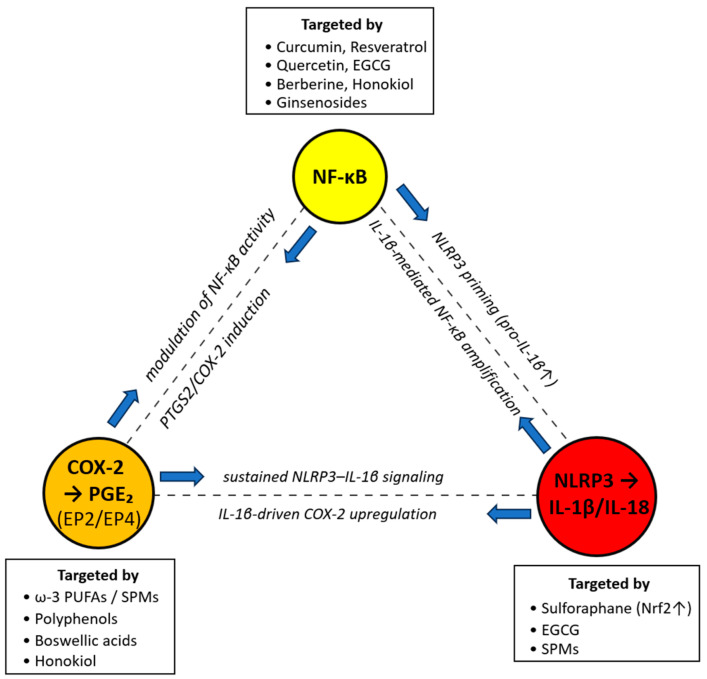
**An inflammatory triangle in glioma: directional crosstalk and natural modulators.** The presented nodes include NF-κB (yellow), COX-2→PGE_2_ (EP2/EP4) (orange), and NLRP3→IL-1β/IL-18 (red). Blue arrows on dashed edges indicate direction of effect with the mechanisms labeled along each edge: (1) NF-κB→NLRP3: NLRP3 priming (pro-IL-1β ↑); (2) NLRP3 → NF-κB: IL-1β-mediated NF-κB amplification; (3) NF-κB→COX-2/PGE_2_: PTGS2/COX-2 induction; (4) COX-2/PGE_2_→NF-κB: EP2/EP4-dependent NF-κB modulation; (5) NLRP3→COX-2/PGE_2_: IL-18-driven COX-2 upregulation; (6) COX-2/PGE_2_→NLRP3: sustained NLRP3–IL-18 signaling. “Targeted by” boxes list representative natural modulators for each node. Abbreviations: PTGS2 = gene encoding COX-2; PGE_2_ = prostaglandin E_2_; EP2/EP4 = PGE_2_ receptors; SPMs = specialized pro-resolving mediators.

The three above-discussed pathways may take part in several positive-feedback loops; therefore their crosstalk is discussed as an inflammatory triangle as presented in Figure 1.

NF-κB transcriptionally primes NLRP3 and pro-IL-1β, positioning canonical inflammatory signals such as TNFα or TLR ligands to sensitize GBM/TME cells to inflammasome activation. In this context, inflammasome engagement may enhance cytokine release and inflammatory crosstalk, reinforcing the chronic pro-tumorigenic characteristics of the GBM microenvironment [38].

In turn, IL-1β upregulates COX-2, and PGE_2_ (via EP4) reinforces IL-1β producing TAMs and forming an autocrine/paracrine loop that sustains immunosuppression. The signaling between cytokine and eicosanoid pathways preserves chronic inflammation and promotes GBM progression by limiting effective antitumor immunity [53].

PGE_2_ can feed back on NF-κB-regulated genes and myeloid polarization, while NF-κB drives PTGS2 expression, closing a recurring triangle of activation. This interlinked network amplifies inflammatory signaling and reinforces immunosuppressive TAM phenotypes within the GBM microenvironment [38,46]. Because TAMs are among the most abundant immune cells in GBM, often even dominating the leukocyte compartment, these feedback loops may scale system-wide, influencing glioma stem-like niche, angiogenesis, and even therapy response [14]. Single-cell and spatial studies continue to analyze the relative contributions of microglia and infiltrating macrophages, showing that both exhibit context-dependent plasticity strongly influenced by NF-κB, PGE_2_/EP4, and IL-1 signaling. This convergence underscores how inflammatory circuits coordinate innate immune adaptation and immunosuppressive niche formation in GBM [14,54].

In total, these three core components of inflammatory response can extensively interact and influence each other, tipping the balance to an oncogenic microenvironment. The core interactions are presented in Figure 1 as a core inflammatory triangle. The targeted action on antiglioma agents of each component as seen in the figure are presented in the next sections.

### 2.5. Cell-Death and Stress Programs Relevant to Resistance

Beyond cytokine signaling, glioma responses involve apoptosis, autophagy, ferroptosis, and mitochondrial stress programs that interface with NF-κB/COX-2/NLRP3 pathways. For example, ginsenosides can trigger ferroptosis in GBM models [55], while berberine engages mitochondrial apoptosis alongside NF-κB suppression [56], illustrating how anti-inflammatory agents intersect resistance biology. These mechanisms are elaborated per compound class in Section 4.

## 3. Tumor Microenvironment (TME)

Glioma grows within a densely interactive environment of immune, glial, and vascular cells that collectively enforce immune evasion and treatment resistance. Tumor-associated macrophages and microglia (TAMs) are the dominant immune population—frequently approaching ~50% of live cells, a feature underscoring their central role in shaping the TME [14]. Contemporary studies converge on the view that the glioma’s TME may impact the failure of immunotherapies by sustaining chronic, multilayered immunosuppression [37].

Tumor-associated macrophages in glioma comprise two major populations: brain-resident microglia and infiltrating monocyte-derived macrophages (MDMs). These compartments differ in developmental origin, molecular markers, spatial distribution, and function. Microglia, characterized by markers such as TMEM119 and P2RY12, are broadly distributed throughout the tumor and often predominate numerically. In contrast, CCR2^+^ MDMs originate from circulating bone marrow-derived monocytes and tend to accumulate in perivascular and hypoxic regions, where they adopt distinct transcriptional and functional states [57]. Single-cell analyses have revealed distinct macrophage specializations within GBM, including hypoxic, phagocytic/lipid-rich, and transitional states, challenging the classical M1/M2 dichotomy and highlighting the context-dependent plasticity of these cells. Spatial-omics studies further demonstrate that microglial CD39 often localizes adjacent to tumor-cell CD73, forming purinergic signaling that elevates extracellular adenosine levels and is associated with poor clinical outcomes [57].

Cytokine and chemokine networks may also play roles in recruiting and programming myeloid cells within the GBM microenvironment. GBM cells and associated stromal elements secrete CCL2, CCL7, CXCL12, CSF-1, and VEGF, which collectively attract monocytes and myeloid-derived suppressor cells (MDSCs) while predetermining their differentiation toward immunosuppressive phenotypes [58]. In both murine and human glioma models, concurrent blockade of CCL2 and CCL7 is required to effectively inhibit the trafficking of CCR2^+^/CX3CR1^+^ MDSCs [59]. In parallel, elevated TGF-β signaling originating from both tumor and stromal sources exerts immunosuppressive effects and is associated with aggressive disease biology and poor prognosis [60]. These axes interface with inflammatory circuits that can also include NF-κB and feed into COX-2/PGE_2_-dependent prostaglandin signaling, amplifying myeloid recruitment and polarization [57].

Compared with many extracranial tumors, glioma harbors relatively low effector T-cell infiltration and profound dysfunction of the T-cell population [37]. Regulatory T cells (Tregs) prominently contribute to checkpoint blockade resistance. In experimental models of gliomas, Treg-targeted strategies convert suppressive Tregs into effector-like cells and enhance antitumor immunity [61]. However, in clinical trials, these strategies failed in GBM, probably due to the combined effects of immune-cold tumor biology, myeloid suppression, and TME restriction [37].

Another significant cell type in the TME is reactive astrocytes, which concentrate at tumor margins, remodel extracellular matrix, and shape immune responses via cytokines, complement, and metabolic support to tumor cells [62]. Experimental and review studies show that tumor-associated astrocytes promote an immunosuppressive phenotype and can be required for GBM maintenance, with genetic studies in experimental models illustrating a causal influence [63,64]. GBM’s vascular system is also abnormal and heterogeneous. Endothelial cells (ECs) not only deliver oxygen/nutrients but also provide paracrine signaling and support a niche that sustains glioma stem-like cells (GSCs) and invasion [65]. Recent studies have shown that EC-secreted chemokines, such as IL-8 and CXCL12, promote high-grade glioma invasion and chemotaxis [66]. In parallel, blood–brain/blood–tumor barrier (BBB/BTB) heterogeneity limits drug exposure at infiltrative margins [67]. Complementary approaches, such as the use of nanomedicine or MR-guided ultrasound, attempt to bypass these barriers in GBM [68].

*IDH* status is also a feature that we should take into account in the TME. Adult-type *IDH*-mutant gliomas typically have a TME with fewer tumor-infiltrating lymphocytes and an altered microglia myeloid compartment, while *IDH*-wildtype GBM exhibits higher overall immune infiltration but with stronger immunosuppressive features [69,70].

Overall, the immunobiology of glioma and GBM is associated with inflammatory responses and due to that is linked to COX-2/PGE_2_, NF-κB, and inflammasome/IL-1 signaling pathways. Targeting these interconnected axes may offer an approach to change the TME toward a less suppressive state. Agents that reduce PGE_2_ production or block EP2/EP4 receptors, inhibit NF-κB-dependent cytokine expression, or limit IL-1β release might promote such changes. Strategies combined with standard treatments might also prove useful [48,67].

## 4. Natural Anti-Inflammatory Compounds in Glioma and the TME

Natural compounds are diet- or botanical-derived molecules, like polyphenols, isothiocyanates, terpenes, and alkaloids, that may exert multi-target anti-inflammatory and immunomodulatory effects relevant to glioma biology. For this review, we are focusing on the most studied agents and on compound classes with reproducible anti-inflammatory activity relevant to glioma biology. Our focus is on modulation of NF-κB, COX-2/PGE_2_, and inflammasome/IL-1 signaling, while noting BBB/BTB considerations and basic safety [40,71,72].

### 4.1. Polyphenols

Polyphenols are a broad group of antioxidant compounds found in many plant-based foods that may exert immunomodulatory effects through their ability to regulate redox balance and inflammatory signaling networks. Polyphenols such as curcumin, resveratrol, and EGCG can modulate COX-2/PGE_2_, NF-κB, and inflammasome pathways, offering natural means to combat GBM [73].

Curcumin is a bioactive polyphenol from Curcuma longa that exhibits broad anti-inflammatory, antioxidant, and immunomodulatory activities [74]. Recent reviews conclude that curcumin suppresses NF-κB and COX-2, lowers pro-inflammatory cytokines, and interacts with multiple cancer-relevant pathways such as those of MAPKs [75]. In GBM, systematic and narrative reviews report that curcumin has antitumor effects but emphasize the limitations of bioavailability and BBB limit. Based on the heterogeneous study designs, the data support an adjunct rather than a clear antiglioma effect [76,77].

Notably, curcumin synergizes with irradiation and temozolomide (TMZ) in GBM cell lines, reducing proliferation and increasing G2/M arrest and apoptosis at comparatively low micromolar levels [78]. These radiosensitizing effects have been replicated with different radiation modalities and are consistently attributed to interference with survival pathways that intersect NF-κB and related nodes [79]. A 2024 systematic review and meta-analysis of 24 studies (304 animals) reported a significant overall reduction in glioma tumor growth with curcumin across model types and formulations, supporting on-target antitumor effects in vivo [76]. Earlier mechanistic in vivo work also showed curcumin enhances radiation efficacy and curbs GBM stem-like traits, consistent with its in vitro radiosensitization profile [80]. Clinically, curcumin is generally well tolerated, while the data highlight the need for better pharmacokinetic analysis [81,82]. Given a notable antiplatelet activity, high-dose curcumin may also interact with anticoagulants [83].

Resveratrol is a naturally occurring polyphenol found in grapes, berries, peanuts, and red wine. It has gained attention for its antioxidant, anti-inflammatory, and neuroprotective properties across cancer and neurodegenerative disease contexts [84]. In glioma models, resveratrol can enhance temozolomide (TMZ) responses via STAT3 down-regulation and induce apoptosis when combined with TMZ [85,86]. Clinical reviews support generally acceptable safety, but in vivo antiglioma action remains inconsistent, again favoring an adjunct role to standard treatment options [87]. As with other polyphenols, resveratrol exhibits antiplatelet effects that could raise bleeding risk alongside antithrombotic drugs [88].

Epigallocatechin-3-gallate (EGCG) is the major catechin found in green tea and one of the most studied dietary polyphenols. It exhibits strong antioxidant and anti-inflammatory activities, influencing signaling networks that regulate cell survival, metabolism, and immune responses [89]. EGCG down-regulates IL-1β-induced iNOS/COX-2 by stabilizing IκBα and limiting NF-κB activation [89]. Cancer immunology reviews further summarize EGCG’s anticancer and immunomodulatory actions, though GBM-specific clinical data are few [90]. Safety guidance from systematic reviews shows that >800 mg per day of EGCG and other ingredients found in green tea may also elevate liver enzymes, showing that optimal dosing is essential [91].

Quercetin is a flavonoid widely distributed in fruits, vegetables, and teas, known for its potent antioxidant and anti-inflammatory properties [92]. In glioma and GBM models, quercetin has been shown to inhibit tumor-cell migration and invasion by suppressing the GSK3β/β-catenin/ZEB1 signaling axis [93]. It also induces apoptosis, even in MGMT^+^ GBM cells, through modulation of Wnt/β-catenin and Akt/NF-κB pathways, highlighting its potential to overcome resistance mechanisms [94]. Moreover, quercetin can enhance membrane trafficking of TRAIL receptors, promoting bystander cell killing and amplifying cytotoxic responses [95]. Additional studies suggest that quercetin’s regulation of Rac1–p66Shc signaling and ROS levels further contributes to its antitumor activity [96]. Human safety looks acceptable at moderate doses, with ongoing efforts to improve oral bioavailability [97].

Taken together, polyphenols may regulate inflammation and influence invasion and resistance pathways but face delivery constraints in brain tumors. The bibliography suggests polyphenols as low-toxicity adjuncts to standard therapy. An improvement in bioavailability may further aid their action [71].

### 4.2. Isothiocyanates and Organosulfur Compounds

Isothiocyanates, produced from glucosinolate hydrolysis in cruciferous vegetables, and organosulfur compounds from garlic and related plants are recognized for their potent cytoprotective and anti-inflammatory actions. These phytochemicals influence key regulators such as NF-κB, Nrf2, and inflammasome signaling, linking them to antitumor and immune-modulatory effects [98,99].

A well-studied molecule in this category is sulforaphane (SFN). SFN activates Nrf2 transcription factor, offering cytoprotective action and may also inhibit the NLRP3 inflammasome, directly intersecting inflammation and oxidative stress pathways [100]. In GBM models, SFN has been shown to reverse resistance to TMZ by down-regulating NF-κB and MGMT, and to independently trigger UPR-mediated apoptosis via ATF4–CHOP [17]. Recent studies reinforce SFN’s CNS-relevant anti-inflammatory and neuroprotective profile, supporting its candidacy for TME-targeting strategies [17].

In total, isothiocyanates like SFN demonstrate broad activity, simultaneously activating Nrf2-driven antioxidant responses while suppressing NF-κB and IL-1-mediated inflammatory signaling. In glioma and GBM models, these effects extend to enhanced sensitivity to temozolomide (TMZ), suggesting potential synergy with standard therapy. Their favorable safety profile as dietary metabolites supports further exploration, although optimal dosing, stability, and formulation strategies will be critical to ensure adequate brain exposure and therapeutic efficacy [17,100].

### 4.3. Terpenes, Lignans, and Saponins

Terpenes (including monoterpenes and triterpenoids), lignans, and saponins are structurally distinct phytochemicals found in many herbs, seeds, and medicinal plants. Through modulation of NF-κB, STAT3, and Nrf2 signaling, these compounds demonstrate anti-inflammatory, pro-apoptotic, and immune-regulatory effects relevant to cancer biology [101].

Boswellic acids, especially acetyl-11-keto-β-boswellic acid (AKBA), are non-redox inhibitors of 5-lipoxygenase that also modulate NF-κB signaling, providing a complementary “double hit” on prostanoid and leukotriene pathways [102]. In glioma and GBM models, AKBA exerts antiproliferative effects and influences cellular metabolism and autophagy, indicating potential for therapeutic synergy with existing treatments [103]. Clinically, boswellic acid formulations have been evaluated in small studies for the management of radiation-induced cerebral edema and necrosis, reflecting their anti-inflammatory activity in the CNS, although direct evidence of efficacy against GBM progression remains limited [104].

Ginsenosides, the principal bioactive saponins of Panax species, display diverse anti-inflammatory and antiglioma activities. Specific compounds such as Rg5 inhibit GBM growth through ferroptosis mediated by the NR3C1–HSPB1–NCOA4 axis [55], while RK3 has been shown to suppress glioma phenotypes in both in vitro and in vivo models [105]. Broader reviews highlight the immunomodulatory action of ginsenosides, including inhibition of NF-κB signaling and regulation of endothelial–glial interactions, underscoring a potential in assisting GBM therapy [106].

Honokiol is a biaryl lignan derived from Magnolia species, notable for its ability to cross the blood–brain barrier and accumulate in the CNS, particularly when delivered in liposomal formulations [107,108]. In GBM models, honokiol reprograms tumor-associated macrophages toward pro-inflammatory M1-like states while suppressing tumor growth. It also targets glioma stem-like cells in preclinical studies, and isolated clinical reports describe the use of liposomal honokiol in recurrent GBM, suggesting translational feasibility as an adjunct therapeutic strategy [109].

These compound classes intersect multiple inflammatory and metabolic pathways—including leukotriene signaling, ferroptosis regulation, and macrophage polarization—and may act synergistically with COX-2, NF-κB, and IL-1-targeted strategies. Among them, honokiol and various ginsenosides show particularly promising effects on the TME, highlighting their potential as adjunct modulators of GBM.

### 4.4. Fatty Acids and Specialized Pro-Resolving Mediators (SPMs)

Fatty acids, particularly omega-3 polyunsaturated species, serve as precursors for specialized pro-resolving mediators (SPMs) including resolvins, protectins, and maresins. These lipid mediators orchestrate the active resolution of inflammation through modulation of myeloid cell activity, cytokine clearance, and restoration of tissue integrity [110].

In temozolomide-resistant glioma cells, the omega-3 polyunsaturated fatty acids eicosapentaenoic acid (EPA) and docosahexaenoic acid (DHA) disrupt resistant phenotypes and alter cellular metabolism, supporting the concept that lipid mediators can influence therapy responsiveness [111]. In vivo, DHA has been shown to reduce intratumoral levels of PGE_2_ and prostacyclin, consistent with suppression of COX-2 activity and potential modulation of the TME [112]. Broader oncology and neuro-oncology studies further indicate that omega-3 PUFAs may inhibit tumor progression in part by shifting eicosanoid profiles away from PGE_2_ dominance, although clinical data specific to GBM remain preliminary [113,114].

Specialized pro-resolving mediators (SPMs) such as resolvins, protectins, and maresins actively terminate inflammation rather than simply suppressing it, promoting leukocyte clearance and restoration of tissue homeostasis in preclinical models of cancer and neuroinflammation [115]. Although GBM-specific data remain limited, the mechanistic rationale for integrating ω-3-derived SPMs with standard-of-care therapies is compelling, given their potential to counteract chronic inflammation and immune dysfunction within the GBM TME [116].

By reducing PGE_2_ signaling and enhancing pro-resolving pathways, omega-3 fatty acids and their SPM derivatives align mechanistically with COX-2 and EP-receptor-targeted strategies. In the era of personalized medicine, successful clinical translation is needed to optimize dosing, formulation, and to identify patients most likely to benefit [112,115].

### 4.5. Isoquinoline Alkaloids

Isoquinoline alkaloids are a diverse class of plant-derived compounds with notable bioactive properties, including anti-inflammatory, antioxidant, and antitumor effects. Through modulation of signaling pathways such as NF-κB, MAPK, and apoptotic cascades, these alkaloids have shown potential to influence cancer-cell proliferation, survival, and the TME [117]. Among them, berberine (BBR) consistently inhibits NF-κB signaling, reduces pro-inflammatory cytokines, and triggers apoptosis, with glioma-cell data showing growth and migration suppression through TGF-β/SMAD and mitochondrial pathways [56]. Reviews highlight CNS-protective actions but also underscore low oral bioavailability, reinforcing the need for delivery optimization in brain tumors [118].

### 4.6. Converging Mechanisms

The data presented in Section 4 are summarized in Table 1. Across classes, we have shown that the compounds in this section are associated with NF-κB, COX-2–PGE_2_ (EP2/EP4), and NLRP3–IL-1 inflammatory pathways. Polyphenols (curcumin, resveratrol, EGCG, quercetin) consistently temper NF-κB/COX-2 programs while curbing invasion and, in several models, heightening radio-/chemo-sensitization. Sulforaphane also leads to Nrf2 activation and selective NLRP3 dampening, with signals for reversing TMZ resistance. Terpenes/lignans/saponins (boswellic acids, honokiol, ginsenosides) extend this footprint to leukotriene–NF-κB crosstalk, macrophage re-polarization in the TME, and, in some cases, ferroptosis. Lipid mediators (omega-3 PUFAs and specialized pro-resolving mediators) shift eicosanoid balance away from PGE_2_, and berberine suppresses NF-κB and pro-inflammatory cytokines with additional effects on TGF-β/SMAD and mitochondrial apoptosis. Taken together, these actions point to a multi-node modulation of glioma inflammation and the microenvironment. Real-world use should be formulation-aware and accompanied by safety/interaction checks, with target-engagement biomarkers confirming NF-κB, PGE_2_–EP2/EP4, or IL-1 modulation. Resistance mechanisms are presented in Table 2.

## 5. Conclusions

Chronic, tumor-promoting inflammation is a modifiable co-driver of glioma biology. Across this review we focused on three convergent axes—NF-κB, COX-2/PGE_2_, and the NLRP3–IL-1 pathway—and on how natural anti-inflammatory agents and diet–microbiome strategies can dial down these signals while restructuring the TME to avoid immune tolerance. These interventions have not been presented as stand-alone cures but rather as adjunct and chemopreventive agents that complement surgery, radiotherapy, and chemotherapy with comparatively low toxicity. Our synthesis is narrative and focuses on mechanistic and translational themes rather than on a systematic enumeration of all studies. Many findings are preclinical or observational; human data remain limited and heterogeneous. Effects likely depend on brain exposure/BTB heterogeneity, product formulation/standardization, and patient context (e.g., steroid exposure, metabolic profile). These factors motivate biomarker-anchored, formulation-aware trials to define clinical utility.

We have seen that multiple compounds (e.g., curcumin, resveratrol, quercetin, sulforaphane, honokiol; omega-3-derived mediators) suppress NF-κB/COX-2/IL-1 signaling, reduce invasion, induce apoptosis, and sensitize tumors to radiation or temozolomide. Early human studies show feasibility and symptom-control potential in selected settings, especially as an adjunct strategy, but demonstrating definitive benefit still requires well-designed trials. Mechanistically, the myeloid-dominant TME is critical. Interventions that reduce PGE_2_, inhibit NF-κB-driven cytokine programs, or limit NLRP3/IL-1β output can shift tumor-associated microglia/macrophages away from suppressive states. In total, treating neuroinflammation as a therapeutic opportunity might open a path for low-toxicity, inflammation-targeted adjuncts in glioma. We suggest that by strict translational design and patient-centered implementation, these strategies can be further assessed to draw conclusions on whether they are potent adjunct therapeutic interventions against this devastating disease.

### Future Directions

Priorities include biomarker-anchored trials (NF-κB/COX-2/IL-1 activity and EP receptor tone), validated brain exposure for chosen formulations (addressing BBB/BTB heterogeneity), and combination designs with chemoradiation or targeted agents. Given potential neuroprotective effects and immune system augmentation, future studies should incorporate cognitive outcomes and safety pharmacology tailored to neuro-oncology.

## Figures and Tables

**Table 1:** Synopsis of natural anti-inflammatory compounds in glioma.

Molecule	Class	Primary Inflammatory Targets	GBM-Relevant Actions	Evidence Stage (GBM) *
Curcumin	Polyphenol	NF-κB ↓ COX-2/PGE_2_ ↓	Radiosensitization; chemo-sensitization (TMZ); anti-invasion	in vitro ++; in vivo (orthotopic) +
Resveratrol	Polyphenol (stilbene)	NF-κB crosstalk ↓; COX-2/PGE_2_ ↓ (indirect)	STAT3 ↓; TMZ synergy; anti-migration	in vitro ++; in vivo +
EGCG	Polyphenol (catechin)	NF-κB ↓; COX-2/iNOS ↓; IL-1β signaling ↓	Potentiates combos (e.g., metabolic + TMZ)	in vitro ++; in vivo +
Quercetin	Polyphenol (flavonol)	NF-κB ↓ (indirect); COX-2 pathway ↓	Wnt/β-catenin ↓; anti-invasion; pro-apoptotic	in vitro ++; in vivo +
Sulforaphane	Isothiocyanate	NF-κB ↓; NLRP3/IL-1β ↓	Reverses TMZ resistance (MGMT ↓); ATF4-CHOP apoptosis	in vitro ++; in vivo ++
Boswellic acids (e.g., AKBA)	Triterpenoid acids	5-LOX ↓; NF-κB ↓ (crosstalk); COX-2 cross-regulation	Anti-edema/anti-angiogenic signals	in vitro +; early human (supportive care) ±
Ginsenosides (e.g., Rg5, RK3)	Triterpenoid saponins	NF-κB ↓ (class effect); COX-2 ↓ (reported)	Ferroptosis induction; antiproliferative	in vitro +; in vivo +
Honokiol	Lignan	NF-κB ↓; COX-2/PGE_2_ ↓ (indirect)	TAM re-polarization (M2→M1); GSC targeting; brain-accumulating (liposomal)	in vitro ++; in vivo ++; early human ±
ω-3 PUFAs (EPA/DHA)	Fatty acids	COX-2 substrate shift → PGE_2_ ↓	Pro-resolving mediator precursors; metabolism re-tuning	in vitro +; in vivo +
SPMs	Lipid mediators	Pro-resolution of inflammation; IL-1/TNF programs ↓	Immune “de-escalation” without broad suppression	preclinical +
Berberine	Isoquinoline alkaloid	NF-κB ↓; pro-inflammatory cytokines ↓	TGF-β/SMAD and mitochondrial apoptosis effects	in vitro +; in vivo ±

* The evidence stage is denoted with + or ++, based on the available data in each stage. Abbreviations: TMZ, temozolomide; GSC, glioma stem-like cell; PK, pharmacokinetics; 5-LOX, 5-lipoxygenase.

**Table 2:** Resistance mechanisms and representative compounds (GBM models).

Resistance Mechanism/Pathway	Typical Readouts in GBM Models	Representative Compounds	Evidence Stage (GBM) *
NF-κB-driven survival/inflammation	IκBα loss; NF-κB target genes; cytokines	Curcumin, resveratrol, EGCG, honokiol, berberine	in vitro ++; in vivo +
COX-2–PGE_2_ immunosuppression	COX-2/PGE_2_ ↓; EP2/EP4 modulation	Polyphenols; ω-3 PUFAs/SPMs; boswellic acids; honokiol	in vitro +; in vivo +
NLRP3–IL-1 axis	IL-1β/IL-18 ↓; ASC/caspase-1 modulation	Sulforaphane; EGCG; SPMs	preclinical +
Apoptosis (mitochondrial)	Caspase-3/9; BAX/BCL-2; ΔΨm	Berberine; polyphenols	in vitro +; in vivo ±
Autophagy (context dependent)	LC3-II, p62	Polyphenols (selected reports)	in vitro ±
Ferroptosis	GPX4/ACSL4; lipid-ROS	Ginsenosides (e.g., Rg5)	in vitro +; in vivo +

* The evidence stage is denoted with + or ++, based on the available data in each stage.

## Data Availability

No new data were created or analyzed in this study. Data sharing is not applicable to this article.

## Abbreviations

BBB: blood–brain barrier; BTB: blood–tumor barrier; GBM: glioblastoma; TME: tumor microenvironment; TAMs: tumor-associated microglia/macrophages; PGE_2_: prostaglandin E_2_; EP2/EP4: E-prostanoid receptors 2/4; COX-2: cyclooxygenase-2; NLRP3: NOD-like receptor family pyrin domain-containing 3; SPMs: specialized pro-resolving mediators; PUFAs: polyunsaturated fatty acids; TMZ: temozolomide; GSCs: glioma stem-like cells.
